# Effect of optical correction on choroidal structure in children with anisohypermetropic amblyopia

**DOI:** 10.1371/journal.pone.0231903

**Published:** 2020-04-23

**Authors:** Tomo Nishi, Tetsuo Ueda, Yuutaro Mizusawa, Kentaro Semba, Kayo Shinomiya, Yoshinori Mitamura, Shozo Sonoda, Eisuke Uchino, Taiji Sakamoto, Nahoko Ogata

**Affiliations:** 1 Department of Ophthalmology, Nara Medical University, Nara, Japan; 2 Department of Ophthalmology, Institute of Biomedical Sciences, Tokushima University Graduate School, Tokushima, Japan; 3 Department of Ophthalmology, Kagoshima University Graduate School of Medical and Dental Sciences, Kagoshima, Japan; Sankara Nethralya, Medical Research Foundation, INDIA

## Abstract

The aim of this study was to assess the effect of wearing optical correction on the choroidal structure in eyes of children with anisohypermetropic amblyopia. This study was conducted at the Nara Medical University Hospital and at the Tokushima University Hospital. Twenty-nine anisohypermetropic amblyopic eyes and their fellow eyes of 29 amblyopic patients (mean age, 5.7 ± 1.7 years, range 3- to 8-years) and twenty eyes of 20 age-similar control children (4.9 ± 0.8 years, range 4- to 6-years) were studied. All patients wore optical correction and 15 patients had both optical correction and patching. The values at the baseline were compared to that at one year later. The binarization method was used to determine the total, luminal, and stromal areas of the choroid in the enhanced depth imaging optical coherence tomographic images. The best-corrected visual acuity (BCVA) of the amblyopic eyes was significantly improved after the one-year period. A large luminal area was characteristic of the amblyopic eye at the baseline, and it was significantly reduced after the optical treatment. The stromal area widened significantly in the amblyopic and fellow eyes after one year whereas there were no significant changes in the choroid of the control eyes after one year. After one-year of optical correction, the luminal/stromal ratios in the amblyopic and fellow eyes were decreased and were then not significantly different from that of the normal control eyes. There was a significant and positive correlation between the improvement of the BCVA and the stromal area at the baseline (*r* = 0.64, *P* = 0.001). Wearing corrective lenses on the amblyopic eyes improves the BCVA, and the choroidal structure of the amblyopic eye becomes closer to that of the control eyes. The narrowed luminal area is a specific response of the amblyopic eye associated with the correction of the refractive error. The larger stromal area in the amblyopic eyes at the baseline is a predictive factor for improvements of the BCVA.

## Introduction

Anisohypermetropic amblyopia is a disorder of the visual system that is characterized by a reduction of the visual acuity in one eye in the absence of ocular and neural pathological changes. [1] We recently reported that the retinal microstructures and the choroidal structures in anisohypermetropic amblyopic eyes of children were significantly different from that of normal eyes. [2–4] Furthermore, wearing corrective lenses for one year improved the visual acuity and induced changes of the retinal microstructures in eyes with anisohypermetropic amblyopia. [5] The question then arose as to whether there are structural changes of the choroid after wearing optical correction in children with anisohypermetropic amblyopia.

The choroid plays a significant role in the development of the function and refractive error of the eye, and its development can be influenced by the refractive error. [6–9] There have been several studies on the choroidal thickness of children. [10–15] It has been reported that the subfoveal choroid was thicker in anisohypermetropic amblyopic eyes than that of their fellow eyes and age-matched control eyes. [3,10–15]

Many studies have demonstrated that the visual acuity of amblyopic eyes can be improved by wearing fully corrective lenses. [16,17] However, a consensus on whether the choroidal structure of amblyopic eyes can be changed by an optical treatment has not been conclusively determined. We recently reported that amblyopic and fellow eyes with thicker choroids had a thinning whereas the amblyopic and fellow eyes with thinner choroids had a thickening of the subfoveal choroid by wearing corrective lenses. [5] This report was unique because it demonstrated that wearing fully corrective lenses on amblyopic eyes improved not only the visual acuity but also altered the subfoveal choroidal thicknesses. [5] Moreover, we reported that the choroidal structure in the amblyopic eyes was different from in the control eyes. [4] However, whether optical treatment will alter the choroidal structures has not been determined.

Thus, the aim of this study was to assess whether the choroidal structures are changed by wearing correction optical lenses in children with anisohypermetropic amblyopia. The choroidal structures were determined by optic coherence tomography (OCT) using the binarization method.

## Materials and methods

### Patients and controls

After receiving approval from the Internal Review Board of the Nara Medical University and the Tokushima University, this study was performed from April 2012 to September 2018 at the Nara Medical University Hospital and the Tokushima University Hospital. This study was retrospective, comparative, and observational. The protocol of this study adhered to the tenets of the Declaration of Helsinki. A written informed consent was obtained from parents of the patients. A participant was diagnosed as amblyopic when the BCVA in the amblyopic eye was worse than 20/30 and the BCVA was at least two Snellen lines worse than that of the fellow eye. [18] Anisohypermetropia was diagnosed to be present when the refractive error (spherical equivalent) of one eye was ≥1.5 diopters (D) greater than that of the fellow eye. [18] The control group consisted of children whose BCVA was ≥20/20, whose age was from 4 to 6 years, and whose refractive error (spherical equivalent) was -1.12 to +3.75 D. Moreover, they had no ocular disorders in both eyes. We examined the control children at the Nara Medical University Hospital and at a nursery school of the Tokushima University Hospital during a group medical examination. The exclusion criteria were children with organic eye diseases and history of intraocular surgery. In addition, children who were not able to take the optical examinations were excluded.

The following characteristics were reviewed. All participants were examined by the measurements of the BCVA, extraocular motility assessments, slit-lamp examinations, dilated ophthalmoscopy, and OCT. The BCVA was evaluated by a standard Snellen chart. The decimal BCVA was converted to the logarithm of the minimal angle of resolution (logMAR) for the statistical analyses. The spherical equivalents were measured through dilated pupils by an autorefractor/keratometer (KR-8100, RM8900, Topcon, Tokyo, Japan). We used for measuring the axial length the IOL Master (Carl Zeiss Meditec, Dublin, CA) at the Nara Medical University, and the AL-2000 (TOMEY, Nagoya, Japan) at the Tokushima University. All ocular examinations were conducted between 13:00 to 15:00 hours not to affect the diurnal variations in the choroidal thickness. [19]

All OCT images were obtained by an experienced ophthalmologist or by one of the authors. The choroidal area was evaluated by the OCT horizontal images. The choroidal area was measured by the binarized method. This method was performed by an open access software, ImageJ (version 1.50a: NIH, Bethesda, Maryland, USA). The procedure for this was described in detail by Sonoda et al. [20] The measured area of the choroid is shown in Fig 3, and two graders determined choroidal area. They were masked to the other clinical findings and measured the choroidal area independently. The final choroidal area was the arithmetic means of the values measured by the two observers. The inter-observer reproducibility was evaluated by intraclass correlation coefficients (ICCs).

The effects of the optical correction and patching treatment on the choroidal area of anisohypermetropic amblyopic eyes were assessed. All patients had one year of optical treatment, and patching therapy was added after 12 weeks in 18 cases. The optical treatment has been described in detail. [5] We examined the control children at the baseline and after one year without optical treatments.

### Statistical analyses

The data are presented as the means ± standard deviations (SDs). The BCVA, axial length, and refractive error (spherical equivalent) of the amblyopic, fellow, and control eyes were compared by paired *t* tests before and after one year. The luminal, stromal and total choroidal areas of the amblyopic, fellow, and the control eyes were compared by one-way ANOVA at the baseline and after one year. If a significant difference was found by ANOVA, pairwise comparison was performed with the Tukey test. The significance of the correlations between the baseline choroidal area, axial length, refractive errors, changes of the choroidal area, and changes of the visual acuity was determined by Pearson’s correlation coefficient. Associated factors (*P* <0.1) from univariable analysis were included in the multiple linear regression models. The standardized coefficients (β) for each independent variable was calculated. A *P* <0.05 was taken to be significant. We performed the statistical analysis with the SPSS version 21.0; SPSS Inc., Chicago, IL.

## Results

### Baseline demographic data

Twenty-nine amblyopic and fellow eyes of 29 patients with anisohypermetropic amblyopia whose mean ± SD age was 5.7 ± 1.7 years with a range of 3- to 8-years were studied. For controls, twenty eyes of 20 age-similar control children (4.9 ± 0.8 years, range 4 to 6 years) were studied. The amblyopic patients consisted of 12 boys and 17 girls and the control children group consisted of 7 boys and 13 girls. The BCVA, refractive error, and axial length of 22 of these 29 amblyopic patients and 13 controls before treatment have been reported. [2,3] The BCVA, refractive error, and axial length of twenty-four of these 29 amblyopic patients and 13 controls before treatment have been reported. [5] The choroidal area of twenty-four of these 29 amblyopic patients and 13 controls before treatment have also been reported. [4]

The mean BCVA at the baseline was 0.46 ± 0.26 logMAR units in the amblyopic eyes, -0.01 ± 0.08 logMAR units in the fellow eyes, and -0.05 ± 0.05 logMAR units in the control eyes (Tables 1 and 2). The mean refractive error was +4.14 ± 1.92 diopters (D) in the amblyopic eyes and +0.17 ± 1.00 D in the control eyes (Tables 1 and 2). The mean axial length of the amblyopic eyes before the treatment was 21.1 ± 0.6 mm and that of the control eyes was 21.8 ± 0.8 mm (Tables 1 and 2). The luminal area was significantly larger in the amblyopic eyes than in the control eyes at the baseline (*P* = 0.001, ANOVA, Tukey). There was no significant difference in the total and stromal areas between the amblyopic and fellow and control eyes at the baseline (*P* >0.05, ANOVA).

**Table 1:** Data of the amblyopic eye of the amblyopic patients before and after one-year optical treatment.

	Baseline (n = 29)	After treatment (n = 29)	P^1^
**Age**	5.7 ± 1.7		
**Visual Acuity (logMAR)**	0.46 ± 0.26	0.06 ± 0.15	0.001
**Refractive Error (Spherical equivalent) (D)**	+4.14 ± 1.92	+4.13 ± 1.90	0.88
**Axial Length (mm)**	21.1 ± 0.6	21.5 ± 0.6	0.15
**Total Choroidal Area (×10^4^ μm^2^)**	53.8 ± 7.7	49.0 ± 12.6	0.08
**Luminal Choroidal Area (×10^4^ μm^2^)**	39.0 ± 9.3	31.1 ± 7.6	0.001
**Stromal Choroidal Area (×10^4^ μm^2^)**	14.8 ± 4.7	17.9 ± 5.6	0.027
**Luminal/Stromal Ratio**	3.2 ± 2.0	1.8 ± 0.5	0.001

1 paired *t* tests.

Data are expressed as the means ± standard deviations.

**Table 2:** Data of the fellow eye of the amblyopic patients before and after one-year optical treatment.

	Baseline (n = 29)	After treatment (n = 29)	P^1^
**Visual Acuity (logMAR)**	- 0.01 ± 0.08	- 0.02 ± 0.06	0.69
**Refractive Error (Spherical equivalent) (D)**	+1.97 ± 1.50	+1.64 ± 1.41	0.75
**Axial Length (mm)**	21.8 ± 0.8	21.9 ± 0.8	0.84
**Total Choroidal Area (×10^4^ μm^2^)**	47.6 ± 13.6	50.0 ± 12.8	0.50
**Luminal Choroidal Area (×10^4^ μm^2^)**	32.6 ± 9.6	31.9 ± 8.2	0.78
**Stromal Choroidal Area (×10^4^ μm^2^)**	15.0 ± 5.6	18.0 ± 4.7	0.029
**Luminal/Stromal Ratio**	2.3 ± 0.8	1.8 ± 0.1	0.001

1 paired *t* tests.

Data are expressed as the means ± standard deviations.

### Changes after one year

One year after wearing the optical correction, the BCVA improved significantly from 0.46 ± 0.26 logMAR units to 0.06 ± 0.15 logMAR units in the amblyopic eyes (*P* = 0.001; paired *t* test, Table 1). The refractive error did not change significantly in the amblyopic and fellow eyes after the optical treatment (Tables 1 and 2). The BCVA, the refractive error, axial length, and the choroidal area of twenty-four of these 29 amblyopic patients after treatment and 13 controls after one year have been reported. [5]

**Fig 1:**
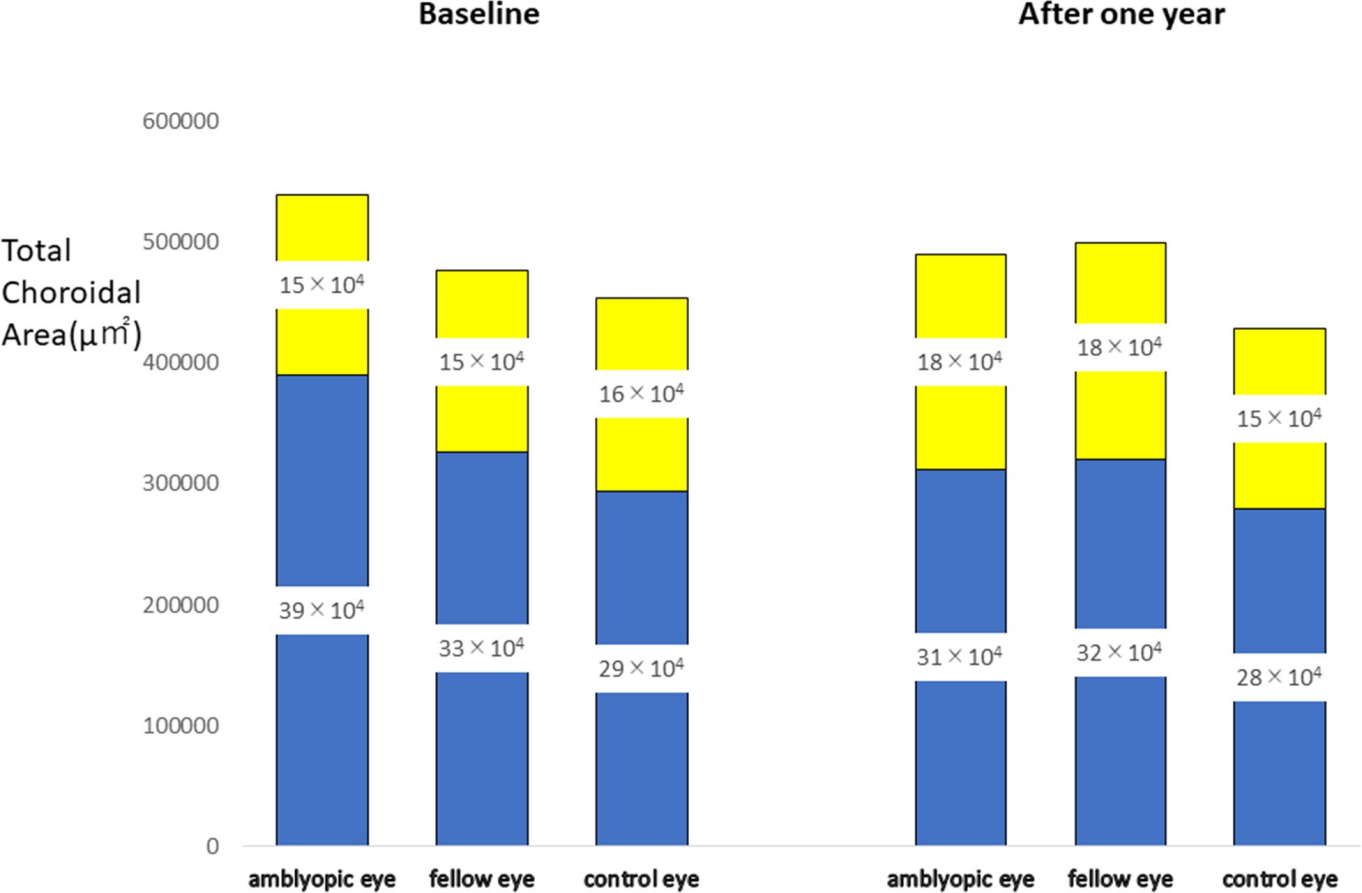
Changes of the choroidal area after one year of optical correction. After one year of the optical correction, the luminal choroidal area in the amblyopic eye was significantly narrowed and the stromal area was significantly widened. In the fellow eyes, the stromal area was significantly widened after the optical correction. There were no significant changes in the control eyes. Blue Area: Luminal Area, Yellow Area: Stromal Area.

One year after the optical correction, the luminal choroidal area in the amblyopic eye was significantly narrowed (*P* = 0.001; paired *t* tests, Table 1 and Fig 1). On the other hand, the stromal choroidal area in the amblyopic eyes was significantly widened (*P* = 0.027; paired *t* test, Table 1 and Fig 1). Thus, the luminal/stromal ratio of the amblyopic eye was significantly decreased after wearing the optical correction compared to that of before the treatment (*P* = 0.001; paired *t* test, Table 1 and Fig 1). The total choroidal area did not change significantly (Table 1 and Fig 1). The differences of the amblyopic, fellow eyes, and control eyes in the total, luminal and stromal areas were not significant (*P* >0.05, ANOVA). In the amblyopic eyes of the children who received only optical correction (n = 11), the luminal choroidal area in the amblyopic eye was significantly narrowed one year after wearing corrective lenses (*P* = 0.03; paired *t* tests, Table 3). In amblyopic eye of the amblyopic children who received optical correction and eye patching (n = 18), the luminal choroidal area in the amblyopic eye was also significantly narrowed (*P* = 0.012; paired *t* tests, Table 3) and the stromal choroidal area in the amblyopic eyes was significantly widened (*P* = 0.034; paired *t* test, Table 3). There was no differential trend between children who received only optical correction and those who received both optical correction and patching.

**Table 3:** The amblyopic eye of the amblyopic patients before and after one-year treatment.

	One-year optical correctionand eye patch therapy(n = 18)			One-year optical correction(n = 11)		
	Baseline	After treatment	P^1^	Baseline	After treatment	P^1^
**Age**	6.1 ± 1.6			5.0 ± 1.6		
**Visual Acuity (logMAR)**	0.43 ± 0.21	0.08 ± 0.17	0.001	0.53 ± 0.32	0.00 ± 0.04	0.001
**Refractive Error (Spherical equivalent) (D)**	+4.56 ± 2.16	+4.41 ± 2.19	0.88	+4.14 ± 1.92	+4.13 ± 1.90	0.63
**Axial Length (mm)**	21.2 ± 0.6	21.4 ± 0.7	0.15	21.1 ± 0.6	21.5 ± 0.6	0.18
**Total Choroidal Area (×10^4^ μm^2^)**	53.1 ± 8.2	49.0 ± 12.3	0.26	55.1 ± 7.0	48.6 ± 13.4	0.17
**Luminal Choroidal Area (×10^4^ μm^2^)**	38.8 ± 9.1	31.2 ± 7.9	0.012	39.4 ± 10.0	30.6 ± 7.7	0.03
**Stromal Choroidal Area (×10^4^ μm^2^)**	14.2 ± 4.0	17.8 ± 5.5	0.034	15.8 ± 5.7	18.0 ± 6.1	0.38
**Luminal/Stromal Ratio**	3.1 ± 2.0	1.8 ± 0.6	0.001	3.2 ± 2.3	1.7 ± 0.3	0.05

1 paired *t* tests.

Data are expressed as the means ± standard deviations.

In the fellow eyes, the stromal area was significantly widened (*P* = 0.029; paired *t* test, Table 2 and Fig 1). Thus, the luminal/stromal ratio of the fellow eye was significantly decreased after wearing the optical correction (*P* = 0.001; paired *t* test, Table 2 and Fig 1). In fellow eye of the amblyopic children who received only optical correction (n = 11), the luminal/stromal ratio was significantly decreased after wearing the optical correction (*P* = 0.03; paired *t* tests, Table 4). In fellow eye of the amblyopic children who received optical correction and eye patching (n = 18), the stromal area was significantly widened (*P* = 0.02; paired *t* test, Table 4). Thus, the luminal/stromal ratio of the fellow eye was significantly decreased after wearing the optical correction (*P* = 0.001; paired *t* test, Table 4). There was no differential trend in the choroidal structures between children who received only optical correction and those who received both optical correction and patching.

**Table 4:** Data of the fellow eye of the amblyopic patients before and after one-year treatment.

	One-year optical correction and eye patch therapy (n = 18)			One-year optical correction (n = 11)		
	Baseline	After treatment	P^1^	Baseline	After treatment	P^1^
**Visual Acuity (logMAR)**	- 0.01 ± 0.09	- 0.02 ± 0.07	0.68	- 0.01 ± 0.08	- 0.04 ± 0.05	0.18
**Refractive Error (Spherical equivalent) (D)**	+2.03 ± 1.76	+1.93 ± 1.45	0.86	+1.86 ± 1.10	+1.18 ± 1.20	0.23
**Axial Length (mm)**	21.9 ± 0.7	21.9 ± 0.8	0.97	21.8 ± 0.7	22.0 ± 0.8	0.61
Total Choroidal Area (×10^4^ μm^2^)	51.0 ± 9.8	54.8 ± 9.8	0.25	42.0 ± 17.3	42.1 ± 13.5	0.98
Luminal Choroidal Area (×10^4^ μm^2^)	34.7 ± 6.4	34.9 ± 6.3	0.89	29.2 ± 12.9	26.9 ± 8.6	0.64
Stromal Choroidal Area (×10^4^ μm^2^)	16.3 ± 5.2	19.9 ± 3.6	0.02	12.9 ± 5.6	15.1 ± 5.0	0.34
**Luminal/Stromal Ratio**	2.3 ± 0.8	1.8 ± 0.1	0.001	2.4 ± 0.9	1.8 ± 0.1	0.03

1 paired *t* tests.

Data are expressed as the means ± standard deviations.

The ocular examinations of the control children were done at the baseline and after one year without optical treatments. In these eyes, there were no significant changes in the choroidal areas after one year (Table 5 and Fig 1).

**Table 5:** Data of the controls of the baseline and after one year.

	Baseline (n = 20)	After one year (n = 20)	P^1^
**Age**	4.9 ± 0.8		
**Visual Acuity (logMAR)**	- 0.05 ± 0.05	- 0.07 ± 0.05	0.21
**Refractive Error (Spherical equivalent) (D)**	+0.17 ± 1.00	- 0.11 ± 1.30	0.47
**Axial Length (mm)**	21.8 ± 0.6	22.0 ± 0.6	0.22
Total Choroidal Area (×10^4^ μm^2^)	45.3 ± 14.3	42.9 ± 11.5	0.55
Luminal Choroidal Area (×10^4^ μm^2^)	29.3 ± 1.0	27.9 ± 8.4	0.63
Stromal Choroidal Area (×10^4^ μm^2^)	15.9 ± 4.7	14.9 ± 3.5	0.43
Luminal/Stromal Ratio	1.8 ± 0.2	1.8 ± 0.3	0.66

1 paired t test.

Data are expressed as the means ± standard deviations.

**Fig 2:**
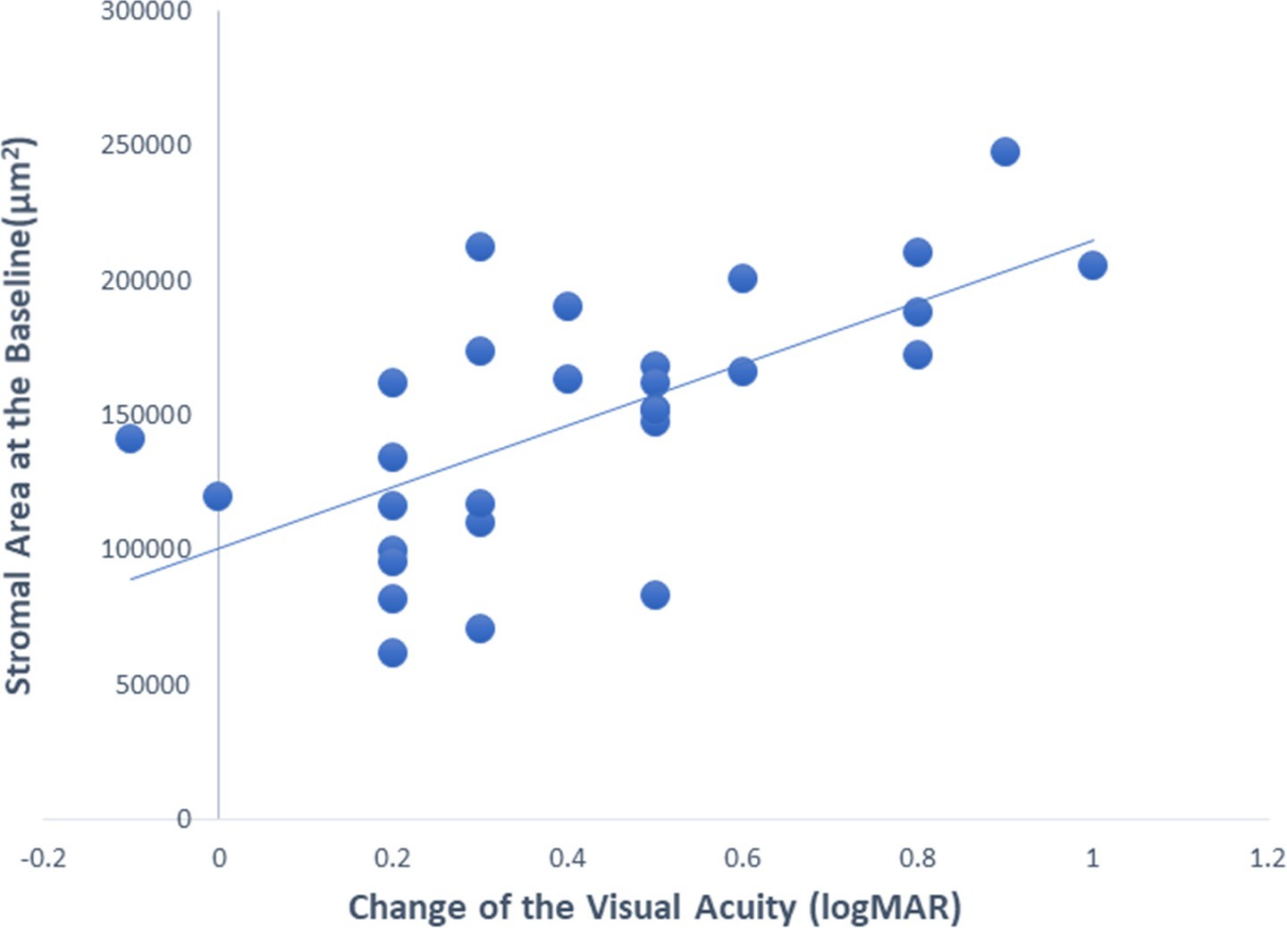
Relationship between the stromal area at the baseline and the changes in the visual acuity in the anisohypermetropic amblyopic eyes. There was a significant positive correlation between the stromal area at the baseline and the change of the visual acuity (*r* = 0.64, *P* = 0.001; Pearson’s correlation coefficient). ◆: amblyopic eyes.

In the univariate analysis of the amblyopic eyes using the structural parameters (total choroidal area, luminal, stromal areas, and luminal/stromal ratio) and the axial length, the improvement of the BCVA was significantly correlated with the stromal area at the baseline (*r* = 0.64, *P* = 0.001; Pearson’s correlation coefficient; Fig 2). There was no significant correlation between the axial length and total choroidal area at the baseline (*r* = -0.01, *P* = 0.64; Pearson’s correlation coefficient). When the associated factors (*P* <0.1) from univariable analysis and potential confounders (age, sex) were included in the multiple linear regression models, a better visual acuity improvement was associated with the larger stromal area at the baseline (β = 0.766; *P* = 0.001; Table 6).

**Table 6:** Multiple linear regression analysis of amblyopic patient (n = 24).

Independent Variable	Improvement of the Visual Acuity (logMAR)	
	Standardized *β*	P value
**Total Choroidal Area At the Baseline (μm^2^)**	**0.230**	**0.180**
**Luminal Choroidal Area At the Baseline (μm^2^)**	**0.023**	**0.217**
**Stromal Choroidal Area At the Baseline(μm^2^)**	**0.766**	**0.001**

The inter-observer reproducibility of the binarization method was very high with ICC = 0.84 for the total area, 0.82 for the luminal area, and 0.81 for the stromal area.

### Representative anisohypermetropic amblyopic patient and control subject

**Fig 3:**
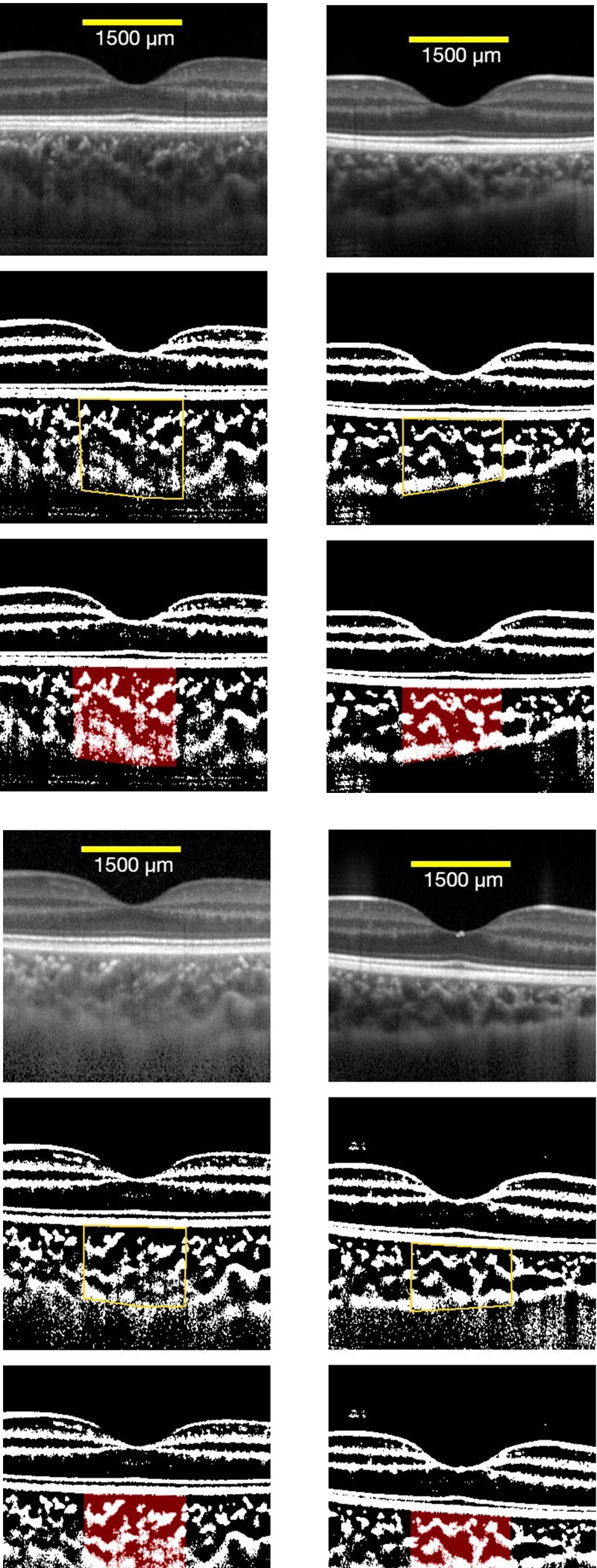
Representative anisohypermetropic amblyopic patient and control subject. The choroidal area was 1500 μm^2^ at 750 μm nasal and 750 μm temporal to the fovea. Vertically, the area measured extended from the retinal pigment epithelium to the chorioscleral border (yellow line). The measured area of the choroid is demarcated (Top). The image is converted to a binary image by the Niblack method by the of ImageJ software (Middle). The dark area which is the luminal area is represented by the red area (Bottom). A: amblyopic eye before the treatment B: control eye at the baseline C: amblyopic eye after the treatment 3D: control eye after one year.

We presented the findings of a representative amblyopic case of a 7-year-old child in Fig 3A and 3B. Our initial examination showed that the BCVA was 0.8 logMAR units in the amblyopic eye, and it improved to 0 logMAR units one year after the beginning of the wearing of the optical correction. At the baseline, the total choroidal area was 55×10^4^ μm^2^, the luminal area was 36×10^4^ μm^2^, the stromal area was 19×10^4^ μm^2^, and the luminal/stromal ratio was 1.9 (Fig 3A). After the one year period of wearing the optical correction, the total and luminal areas were narrowed to 53×10^4^ μm^2^ and 30×10^4^ μm^2^, and the stromal area was widened to 22×10^4^ μm^2^ (Fig 3B) The luminal/stromal ratio was reduced to 1.3.

We presented the findings of a representative 4-year-old control subject in Fig 3C and 3D. At the baseline, the BCVA was 0 logMAR units, the total choroidal area was 48×10^4^ μm^2^, the luminal area was 31×10^4^ μm^2^, stromal area was 17×10^4^ μm^2^, and the luminal/stromal ratio was 1.8 (Fig 3C). After one year, the total, luminal and stromal areas were narrowed to 43×10^4^ μm^2^, 28×10^4^ μm^2^ and 16×10^4^ μm^2^ (Fig 3D).

## Discussion

The results showed that the amblyopic eyes had a larger luminal area than that of control eyes at the baseline. Interestingly, one-year of wearing the optical correction led a significant decrease in the luminal areas only in the amblyopic eyes. On the other hand, the stromal area was widened in both the amblyopic and fellow eyes one year after the treatment. Ruiz-Medrano et al reported that in normal children and adults, the luminal area and the percentage of vascular/total area decreased with increasing age while the stromal area remained stable. [21] They reported that in children (3–10 years), the average percentage of the vascularity was 60.56%. In our cohort, the average vascularity index was comparable at 64.28% (average luminal/stromal ratio,1.8) in the control eyes and 72.72% (average luminal/stromal ratio of 3.2) in the amblyopic eye. The large luminal area at the baseline was characteristic of the amblyopic eye.

The amblyopic eyes with larger stromal areas at the baseline had better improvements of the visual acuity. The stromal area included the nonvascular smooth muscle cells, neurons, vascular walls, and connective tissues. [22] It has been reported that the nonvascular smooth muscle cells of the stromal area are associated with accommodation by modulating the choroidal thickness. [23] In the amblyopic eyes, the accommodation was reduced compared to the fellow eyes. [24] We suggest that the amblyopic eyes with widened stromal areas had more nonvascular smooth muscle cells and had better accommodation. The better accommodation induced the improvement of the visual acuity. [24] Thus, the widened stromal area in both the amblyopic and fellow eyes after treatment is probably a response to the optical correction of the refractive error.

Our findings showed that only the amblyopic eyes had a reduction of the luminal area after the treatment. Thus, this may be a specific response of the amblyopic eye induced by the correction of the refractive error. We recently reported that the choroidal thickness was changed by the optical correction. [5] The change of the subfoveal choroidal thickness in amblyopic and fellow eyes was greater than that in the control eyes. [5] The optical correction probably led to compensatory changes of the subfoveal choroidal thickness in amblyopic and fellow eyes. [5] These changes in the amblyopic and fellow eyes are produced by ocular responses to the correction of the refractive error. However, in another study, it was reported that the choroidal thickness of the amblyopic eyes was decreased after the optical treatment. [25] On the other hand, some studies have reported that the choroidal thickness of the amblyopic eyes was not changed after the optical treatment. [26,27] In these reports the duration of the treatment was different which may explain the differences from our findings.

There are limitations in this study. We investigated a small number of anisohypermetropic amblyopic eyes of Japanese children. Further studies with a larger number of subjects will be necessary to confirm our findings. In addition, we measured the changes after only one year of optical correction, and a longer follow-up period would be desirable.

In conclusion, the narrowing of the luminal area is a specific response in amblyopic eyes produced by correction of the refractive error. The larger stromal area in the amblyopic eyes at the baseline is a predictive factor for improvements of the BCVA.

## Supporting information

S1 Data set (PDF)
