# Supplementary material for: Effect of optical correction on choroidal structure in children with anisohypermetropic amblyopia
Source: PLoS One. 2020 Apr 23;15(4):e0231903. doi: 10.1371/journal.pone.0231903 (PMC7179822; doi:10.1371/journal.pone.0231903)
Supplement: S1 Data set — (PDF) [file pone.0231903.s001.pdf]

Amblyopia

pre

| | Total | luminal | stromal | AXL | VA | SP |
|---|---|---|---|---|---|---|
| 1 | 488026.4 | 371750.3 | 116276.1 | 21.83 | 0.2 | 3 |
| 2 | 490606.8 | 338511.7 | 152095 | 20.9 | 0.5 | 7 |
| 3 | 513488.4 | 379409.7 | 134078.7 | 21.71 | 0.2 | 0.5 |
| 4 | 631573.4 | 419233.8 | 212339.6 | 20.57 | 0.5 | 7.5 |
| 5 | 646825.5 | 536933.7 | 109891.8 | 21.07 | 0.3 | 5 |
| 6 | 545017.5 | 428349.7 | 116667.8 | 21.77 | 0.2 | 3.5 |
| 7 | 582341.5 | 414525.3 | 167816.3 | 22.05 | 0.5 | 5 |
| 8 | 533582.8 | 434210.2 | 99372.68 | 20.77 | 0.2 | 2.75 |
| 9 | 510920.8 | 427897.4 | 83023.48 | 21.02 | 0.5 | 4.5 |
| 10 | 695831.1 | 534009.9 | 161821.2 | 20.91 | 0.5 | 6.5 |
| 11 | 562949.4 | 481415.7 | 81533.7 | 21.54 | 0.2 | 5.25 |
| 12 | 678017.5 | 616074.3 | 61943.17 | 20.62 | 0.2 | 3 |
| 13 | 533090 | 437681.3 | 95408.66 | 22.51 | 0.2 | 1 |
| 14 | 470411 | 350599.1 | 119811.9 | 20.56 | 0.2 | 5.75 |
| 15 | 359578.6 | 218202.1 | 141376.5 | 21.04 | 0.5 | 5 |

| | | | | | | |
|---|---|---|---|---|---|---|
| 16 | 547583.2 | 400606.2 | 146977 | 21.98 | 0.5 | 4.25 |
| 17 | 505872.3 | 342879.6 | 162992.7 | 20.86 | 0.5 | 6 |
| 18 | 562745.9 | 372610.8 | 190135.1 | 21 | 0.8 | 7 |
| 19 | 548568.7 | 360599.9 | 187968.8 | 20.69 | 0.8 | 3.25 |
| 20 | 518083.1 | 312741 | 205342.1 | 20.96 | 1 | 2 |
| 21 | 483768.5 | 310403.8 | 173364.8 | 20.79 | 0.3 | 3.5 |
| 22 | 641039.4 | 393276 | 247763.4 | 21.46 | 1 | 1.5 |
| 23 | 495866.7 | 334061.4 | 161805.3 | 20.88 | 0.2 | 3.75 |
| 24 | 538531.8 | 328631.1 | 209900.8 | 20.32 | 0.8 | 2.75 |
| 25 | 527547.5 | 327160.2 | 200387.2 | 20.46 | 0.6 | 4.125 |
| 26 | 452518 | 280588 | 171930 | 20.54 | 0.8 | 7 |
| 27 | 449271.7 | 297988.6 | 151283.1 | 22.13 | 0.6 | 7.5 |
| 28 | 445149.8 | 279151.2 | 165998.6 | 21.17 | 0.6 | 2.5 |
| 29 | 656444.4 | 585819.2 | 70625.2 | 21.41 | 0.2 | 3.75 |

| Amblyopia post | Total | luminal | stromal | AXL | VA | SP |
|---|---|---|---|---|---|---|
| 1 | 467906.1 | 311038.3 | 156867.8 | 21.95 | 0 | 3 |
| 2 | 433519.2 | 321881.3 | 111637.8 | 21.19 | 0 | 7 |

| | | | | | | |
|---|---|---|---|---|---|---|
| 3 | 594034 | 396450.8 | 197583.2 | 22.13 | 0 | 0.5 |
| 4 | 726359.3 | 449060.3 | 277299 | 20.85 | 0.2 | 7.5 |
| 5 | 455886.4 | 285929.3 | 169957.1 | 21.24 | 0 | 5 |
| 6 | 478693.4 | 287134.5 | 191558.9 | 21.9 | -0.1 | 3.5 |
| 7 | 492420.6 | 298389 | 194031.7 | 21.86 | 0 | 5 |
| 8 | 385542.7 | 242309.1 | 143233.7 | 21.04 | 0 | 2.75 |
| 9 | 317742.3 | 196651.3 | 121091 | 21.19 | 0 | 4.5 |
| 10 | 525135.7 | 307313.3 | 217822.4 | 20.95 | 0 | 6.5 |
| 11 | 430195.5 | 259458.4 | 170737.1 | 21.7 | 0 | 5.25 |
| 12 | 600234.8 | 387659.2 | 212575.6 | 20.6 | 0 | 3 |
| 13 | 637331.8 | 408292.1 | 229039.7 | 22.89 | 0 | 0.5 |
| 14 | 350658.1 | 222682.4 | 127975.8 | 20.29 | 0.2 | 5.75 |
| 15 | 315600 | 254051.7 | 61548.32 | 22.13 | 0.6 | 7.5 |
| 16 | 418229.2 | 274876.9 | 143352.3 | 22.14 | 0 | 4.25 |
| 17 | 557019.4 | 345562.3 | 211457.1 | 21 | 0.1 | 5 |
| 18 | 416717.2 | 261727.6 | 154989.7 | 22.25 | 0.4 | 7 |
| 19 | 530936.8 | 303917 | 227019.7 | 20.84 | 0 | 3.5 |

| | | | | | | |
|---|---|---|---|---|---|---|
| 20 | 644898.6 | 379226.2 | 265672.4 | 21.13 | 0 | 2.25 |
| 21 | 531472.3 | 320281.3 | 211191.1 | 20.93 | 0 | 3.75 |
| 22 | 774571 | 465127.8 | 309443.2 | 22.14 | 0.1 | 2 |
| 23 | 693233.8 | 457082.8 | 236151 | 21.03 | 0 | 3.325 |
| 24 | 455847.7 | 288830.8 | 167016.9 | 20.64 | 0 | 3.25 |
| 25 | 422483 | 280203.2 | 142279.9 | 21.21 | 0 | 2.25 |
| 26 | 250780.1 | 158580.1 | 92200.01 | 20.98 | 0 | 5.75 |
| 27 | 395943.4 | 254232.9 | 141710.5 | 21.05 | 0.1 | 4 |
| 28 | 371387.2 | 227148.2 | 144239 | 21.47 | 0 | 2.5 |
| 29 | 496018.8 | 344248.7 | 151770.1 | 21.87 | -0.1 | 2 |
| Fellow pre | Total | luminal | stromal | AXL | VA | SP |
| 1 | 425724.3 | 307624 | 118100.3 | 22 | -0.1 | 2 |
| 2 | 456979.3 | 350080.8 | 106898.4 | 21.34 | 0 | 5 |
| 3 | 505894.1 | 329697.5 | 176196.6 | 21.83 | 0 | 0 |
| 4 | 495526.5 | 329988.1 | 165538.4 | 21.25 | -0.1 | 5 |
| 5 | 682427.9 | 425853.2 | 256574.7 | 21.74 | 0 | 2.25 |
| 6 | 514346.6 | 421615.5 | 92731.05 | 22 | 0 | 2 |

| | | | | | | |
|---|---|---|---|---|---|---|
| 7 | 468787.4 | 331851.2 | 136936.2 | 23.06 | -0.1 | 0 |
| 8 | 507969.8 | 392857.1 | 115112.6 | 21.21 | -0.1 | 1 |
| 9 | 500364.3 | 350273.7 | 150090.6 | 21.69 | 0.2 | 2 |
| 10 | 510569.7 | 409630.6 | 100939.1 | 21.35 | 0 | 3.5 |
| 11 | 630117.6 | 448363.4 | 181754.2 | 22.8 | 0 | 1 |
| 12 | 574696.2 | 393583.2 | 181113 | 21.03 | 0 | 2 |
| 13 | 522992.4 | 397952.2 | 125040.2 | 22.71 | 0 | 0.75 |
| 14 | 361027.9 | 226209.6 | 134818.2 | 20.64 | 0 | 3 |
| 15 | 432685.7 | 266035.2 | 166650.5 | 21 | 0.2 | 4.5 |
| 16 | 380941.4 | 258408.9 | 122532.5 | 22.98 | 0 | 0 |
| 17 | 435334.7 | 292614.7 | 142720 | 22.36 | 0 | 0.5 |
| 18 | 550901.1 | 340994 | 209907.1 | 22.99 | -0.1 | 4.75 |
| 19 | 606955 | 374805.6 | 232149.4 | 21.52 | 0 | 3.25 |
| 20 | 602474.1 | 453159.5 | 149314.6 | 21.21 | 0 | 1.5 |
| 21 | 425584.6 | 262010.7 | 163573.9 | 21.78 | 0 | 1.5 |
| 22 | 732703.6 | 438454.6 | 294249 | 22.07 | 0 | 0.5 |
| 23 | 520263.6 | 339971.4 | 180292.2 | 21.92 | 0 | 1 |
| 24 | 469934.8 | 296728.4 | 173206.4 | 20.32 | -0.1 | 2.75 |

| | | | | | | |
|---|---|---|---|---|---|---|
| 25 | 261918.7 | 162244.1 | 99674.62 | 21.48 | -0.1 | 2.5 |
| 26 | 132070.8 | 92269.58 | 39801.19 | 22.56 | 0.2 | 1.75 |
| 27 | 261592 | 162802.4 | 98789.63 | 22.66 | -0.1 | 0.5 |
| 28 | 221293.9 | 168092.7 | 53201.2 | 22.15 | 0 | 1 |
| 29 | 612581.9 | 431339 | 181243 | 22.11 | 0 | 1.5 |
| Fellow pre | Total | luminal | stromal | AXL | VA | SP |
| 1 | 425724.3 | 307624 | 118100.3 | 22 | -0.1 | 2 |
| 2 | 456979.3 | 350080.8 | 106898.4 | 21.34 | 0 | 5 |
| 3 | 505894.1 | 329697.5 | 176196.6 | 21.83 | 0 | 0 |
| 4 | 495526.5 | 329988.1 | 165538.4 | 21.25 | -0.1 | 5 |
| 5 | 682427.9 | 425853.2 | 256574.7 | 21.74 | 0 | 2.25 |
| 6 | 514346.6 | 421615.5 | 92731.05 | 22 | 0 | 2 |
| 7 | 468787.4 | 331851.2 | 136936.2 | 23.06 | -0.1 | 0 |
| 8 | 507969.8 | 392857.1 | 115112.6 | 21.21 | -0.1 | 1 |
| 9 | 500364.3 | 350273.7 | 150090.6 | 21.69 | 0.2 | 2 |
| 10 | 510569.7 | 409630.6 | 100939.1 | 21.35 | 0 | 3.5 |
| 11 | 630117.6 | 448363.4 | 181754.2 | 22.8 | 0 | 1 |

| | | | | | | |
|---|---|---|---|---|---|---|
| 12 | 574696.2 | 393583.2 | 181113 | 21.03 | 0 | 2 |
| 13 | 522992.4 | 397952.2 | 125040.2 | 22.71 | 0 | 0.75 |
| 14 | 361027.9 | 226209.6 | 134818.2 | 20.64 | 0 | 3 |
| 15 | 432685.7 | 266035.2 | 166650.5 | 21 | 0.2 | 4.5 |
| 16 | 380941.4 | 258408.9 | 122532.5 | 22.98 | 0 | 0 |
| 17 | 435334.7 | 292614.7 | 142720 | 22.36 | 0 | 0.5 |
| 18 | 550901.1 | 340994 | 209907.1 | 22.99 | -0.1 | 4.75 |
| 19 | 606955 | 374805.6 | 232149.4 | 21.52 | 0 | 3.25 |
| 20 | 602474.1 | 453159.5 | 149314.6 | 21.21 | 0 | 1.5 |
| 21 | 425584.6 | 262010.7 | 163573.9 | 21.78 | 0 | 1.5 |
| 22 | 732703.6 | 438454.6 | 294249 | 22.07 | 0 | 0.5 |
| 23 | 520263.6 | 339971.4 | 180292.2 | 21.92 | 0 | 1 |
| 24 | 469934.8 | 296728.4 | 173206.4 | 20.32 | -0.1 | 2.75 |
| 25 | 261918.7 | 162244.1 | 99674.62 | 21.48 | -0.1 | 2.5 |
| 26 | 132070.8 | 92269.58 | 39801.19 | 22.56 | 0.2 | 1.75 |
| 27 | 261592 | 162802.4 | 98789.63 | 22.66 | -0.1 | 0.5 |
| 28 | 221293.9 | 168092.7 | 53201.2 | 22.15 | 0 | 1 |
| 29 | 612581.9 | 431339 | 181243 | 22.11 | 0 | 1.5 |

| Fellow post | Total | luminal | stromal | AXL | VA | SP |
|---|---|---|---|---|---|---|
| 1 | 496913 | 319097.1 | 177815.9 | 20.21 | -0.1 | 2 |
| 2 | 502950.6 | 321089 | 181861.6 | 21.68 | 0 | 4.5 |
| 3 | 521700.4 | 335367.3 | 186333.1 | 22.13 | 0 | 0 |
| 4 | 519156.6 | 317978.5 | 201178.1 | 21.55 | -0.1 | 4 |
| 5 | 648265.7 | 418487.9 | 229777.8 | 22.12 | 0 | 2 |
| 6 | 611687.8 | 387338.6 | 224349.2 | 22.1 | 0 | 1.75 |
| 7 | 510134.9 | 324764 | 185370.8 | 23.03 | -0.1 | 0 |
| 8 | 516895.7 | 331512.1 | 185383.6 | 21.4 | -0.1 | 1 |
| 9 | 562662.3 | 348146.5 | 214515.8 | 21.95 | 0.2 | 2 |
| 10 | 644853.4 | 409296 | 235557.4 | 21.43 | 0 | 3 |
| 11 | 632484.4 | 410798.1 | 221686.3 | 23.09 | 0 | 1 |
| 12 | 654658.4 | 392476 | 262182.3 | 20.94 | 0 | 2 |
| 13 | 498773.5 | 313802.7 | 184970.8 | 23.01 | 0 | 0.75 |
| 14 | 365119.9 | 238821.3 | 126298.6 | 20.67 | 0 | 2.75 |
| 15 | 427122.1 | 265567.6 | 161554.5 | 21.2 | 0 | 4.24 |

| | | | | | | |
|---|---|---|---|---|---|---|
| 16 | 381079.5 | 249302.8 | 131776.7 | 22.91 | 0 | 0 |
| 17 | 651598.9 | 414590.5 | 237008.4 | 22.55 | 0 | 0.5 |
| 18 | 509344.8 | 321322.2 | 188022.6 | 22.71 | 0 | 3.5 |
| 19 | 549527.5 | 354425.2 | 195102.2 | 21.62 | 0 | 3.5 |
| 20 | 477007.1 | 314000.8 | 163006.3 | 21.32 | 0 | 1.25 |
| 21 | 480600.6 | 314125.9 | 166474.8 | 21.92 | 0 | 1 |
| 22 | 683068.3 | 451422.5 | 231645.7 | 22.14 | 0 | 1 |
| 23 | 673854.1 | 445889.6 | 227964.5 | 21.68 | -0.1 | 2.5 |
| 24 | 468762.9 | 304902.2 | 163860.6 | 20.62 | -0.1 | 2.5 |
| 25 | 346642.6 | 212454.1 | 134188.5 | 22.1 | -0.1 | 0 |
| 26 | 196361.1 | 124927.1 | 71434.01 | 22.54 | -0.1 | 0.25 |
| 27 | 270629.9 | 177800.1 | 92829.8 | 22.64 | -0.1 | 0 |
| 28 | 274187.8 | 184041.7 | 90146.08 | 21.91 | 0 | 1 |
| 29 | 418442.6 | 259565.6 | 158877 | 22.44 | -0.1 | -0.25 |
